# Interaction network of tobacco etch potyvirus NIa protein with the host proteome during infection

**DOI:** 10.1186/s12864-016-2394-y

**Published:** 2016-02-01

**Authors:** Fernando Martínez, Guillermo Rodrigo, Verónica Aragonés, Marta Ruiz, Iris Lodewijk, Unai Fernández, Santiago F. Elena, José-Antonio Daròs

**Affiliations:** Instituto de Biología Molecular y Celular de Plantas (Consejo Superior de Investigaciones Científicas - Universidad Politécnica de Valencia), Avenida de los Naranjos s/n, 46022 Valencia, Spain; The Santa Fe Institute, Santa Fe, NM USA

**Keywords:** Host-virus systems biology, Protein interaction network, RNA virus, Plant virus, *Potyvirus*, Nuclear inclusion *a* protein, Affinity purification mass spectrometry, *Arabidopsis thaliana*

## Abstract

**Background:**

The genomes of plant viruses have limited coding capacity, and to complete their infectious cycles, viral factors must target, direct or indirectly, many host elements. However, the interaction networks between viruses and host factors are poorly understood. The genus *Potyvirus* is the largest group of plus-strand RNA viruses infecting plants. Potyviral nuclear inclusion a (NIa) plays many roles during infection. NIa is a polyprotein consisting of two domains, viral protein genome-linked (VPg) and protease (NIaPro), separated by an inefficiently utilized self-proteolytic site. To gain insights about the interaction between potyviral NIa and the host cell during infection, we constructed *Tobacco etch virus* (TEV, genus *Potyvirus*) infectious clones in which the VPg or the NIaPro domains of NIa were tagged with the affinity polypeptide Twin-Strep-tag and identified the host proteins targeted by the viral proteins by affinity purification followed by mass spectrometry analysis (AP-MS).

**Results:**

We identified 232 different *Arabidopsis thaliana* proteins forming part of complexes in which TEV NIa products were also involved. VPg and NIaPro specifically targeted 89 and 76 of these proteins, respectively, whereas 67 proteins were targeted by both domains and considered full-length NIa targets. Taking advantage of the currently known *A. thaliana* interactome, we constructed a protein interaction network between TEV NIa domains and 516 host proteins. The most connected elements specifically targeted by VPg were G-box regulating factor 6 and mitochondrial ATP synthase δ subunit; those specifically targeted by NIaPro were plasma membrane aquaporin PIP2;7 and actin 7, whereas those targeted by full-length NIa were heat shock protein 70–1 and photosystem protein LHCA3. Moreover, a contextualization in the global *A. thaliana* interactome showed that NIa targets are not more connected with other host proteins than expected by chance, but are in a position that allows them to connect with other host proteins in shorter paths. Further analysis of NIa-targeted host proteins revealed that they are mainly involved in response to stress, metabolism, photosynthesis, and localization. Many of these proteins are connected with the phytohormone ethylene.

**Conclusions:**

Potyviral NIa targets many host elements during infection, establishing a network in which information is efficiently transmitted.

**Electronic supplementary material:**

The online version of this article (doi:10.1186/s12864-016-2394-y) contains supplementary material, which is available to authorized users.

## Background

Plant viruses pack many functions in a few genes and, as a consequence, most viral proteins must target numerous host factors, pathways and structures to propel infection. However, the interaction networks between viral factors and host elements during plant infection are largely unknown [1, 2], particularly if we consider that the number of targets of viral factors in the infected plant cells may be unexpectedly high, as recently shown for some RNA viruses infecting human cells [3, 4]. The goal of this work was to inquire about the complexity of the interaction network established between an essential protein from a plant RNA virus, the nuclear inclusion a (NIa) protein of potyviruses, and the host proteins by means of a high-throughput proteomics approach, affinity purification coupled to mass spectrometry (AP-MS) [5, 6].

Potyviruses (genus *Potyvirus*, family *Potyviridae*) form one of the largest groups of viruses infecting plants and cause important losses in crops worldwide. Their genome consists of a single (+)-strand RNA molecule of about 10,000 nucleotides that is translated in two alternative polyproteins (depending on a frameshift in P3 cistron), which are processed by viral proteases in, apparently, a total of eleven mature products [7]. NIa is a crucial cistron in the potyviral genome. It encodes the NIa protein that consists of two different domains, an amino-terminal viral protein genome-linked (VPg) and a carboxy-terminal protease (NIaPro). VPg is a multifunctional protein involved, at least, in virus replication [8], translation [9] and movement [10]. Notably, VPg remains covalently linked to the 5′ terminus of the virus genomic RNA through a Tyr residue [11] and recruits host translation initiation factor eIF4E, or its isoform eIF(iso)4E, in an interaction that is crucial for infection [12]. NIaPro is a serine protease that specifically recognizes seven-amino-acid-long conserved motifs in the viral polyprotein and, in *cis* and in *trans*, cleaves them between positions 6 and 7 to produce most viral protein products [13]. NIaPro also displays RNA binding activity [14], interacts with the viral RNA-dependent RNA polymerase (NIb protein) and is involved in viral replication [15]. The VPg and the NIaPro domains of NIa are separated by an inefficiently utilized autoproteolytic site [16], which implies that substantial amounts of full-length NIa, and processed VPg and NIaPro products coexist in the infected cells at any moment of the infection process. An additional, even more suboptimal, autoproteolytic site exists in some potyviruses close to the carboxy-terminal end of NIaPro producing additional NIa-derived species in infected tissue that may be functionally relevant [17, 18].

**Fig. 1:**
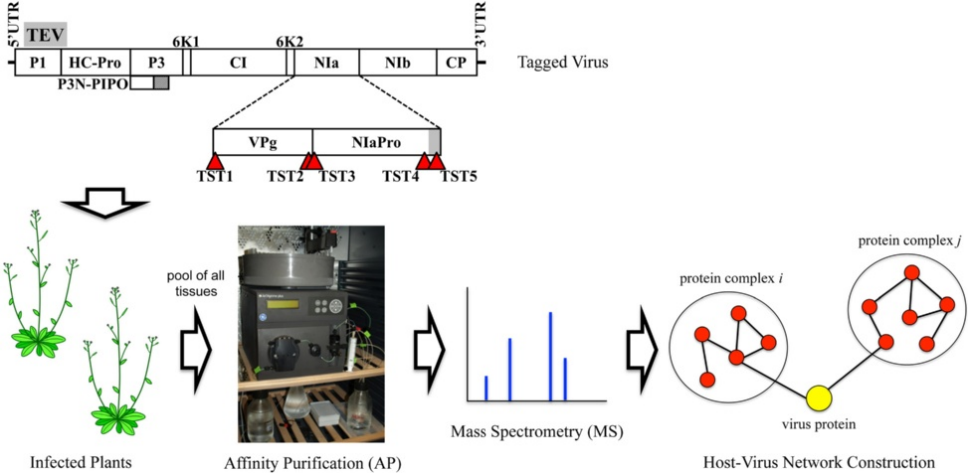
Schematic representation of the TEV recombinant clones in which the NIa protein was tagged with the Twin-Strep-tag (TST) at five different positions (TEV-TSTNIa1 to TEV-TSTNIa5) and affinity purification (AP) mass spectrometry (MS) workflow to construct NIa-host interaction networks. In virus scheme, black lines represent TEV 5′ and 3′ UTR, as indicated. Boxes represent TEV cistrons (P1, HC-Pro, P3, P3N-PIPO, 6K1, CI, 6K2, NIa, VPg, NIaPro, NIb and CP) as indicated. Positions where TST was inserted in the VPg and NIaPro domains of NIa are indicated with red triangles. The gray rectangle in NIaPro represents the carboxy-terminal polypeptide that is subjected to processing through a suboptimal autoproteolytic site

To gain new insights about the complexity of the interaction network between *Tobacco etch virus* (TEV, genus *Potyvirus*) NIa protein and the infected cells, in this work we aimed at identifying host proteins involved in complexes in which NIa or NIa-processing products were engaged during infection by AP-MS. First, we tagged the VPg and NIaPro domains of NIa with an affinity polypeptide while maintaining TEV infectivity. Next, we purified protein complexes from infected tissue by affinity chromatography in native conditions. Then, we identified proteins being part of these complexes by mass spectrometry analysis. Finally, we obtained a series of interaction networks between NIa domains and host elements by computational analysis (Fig. 1).

## Results and discussion

### Construction of infectious TEV clones with tagged NIa

On the basis of the binary plasmid pGTEVat containing an infectious TEV clone under the control of *Cauliflower mosaic virus* (CaMV) 35S promoter and terminator, we constructed five recombinant TEV clones in which the polypeptide Twin-Strep-tag (TST) was inserted at five different positions of NIa (Fig. 1). TST is a bidentate polypeptide that binds Strep-Tactin (a derivative of streptavidin) with high affinity and allows purification of intact protein complexes in mild conditions [19]. We have previously used this tag to successfully purify protein complexes including other tagged TEV proteins from plant infected tissue in native conditions [20]. In TEV-TSTNIa1, TST was inserted after the first three amino-terminal codons of VPg. The position was selected to avoid disruption of the 6K2/VPg cleavage site. These first three codons were repeated after the tag but including a silent mutation to avoid undesired recombination during virus replication. The exact cDNA sequence of the five tagged NIa is in Additional file 1. In TEV-TSTNIa2, TST was inserted between the −9 and −8 codons respect to the VPg/NIaPro cleavage site, again to avoid disturbing this cleavage site. In TEV-TSTNIa3, TST was inserted after the first three codons of NIaPro that, as explained above, were repeated including a silent mutation after the tag. In TEV-TSTNIa4, TST was inserted between the −8 and −7 codons of the internal NIaPro cleavage site. Finally, in TEV-TSTNIa5, TST was inserted between the −9 and −8 codons of NIaPro/NIb cleavage site (Fig. 1 and Additional file 1).

**Fig. 2:**
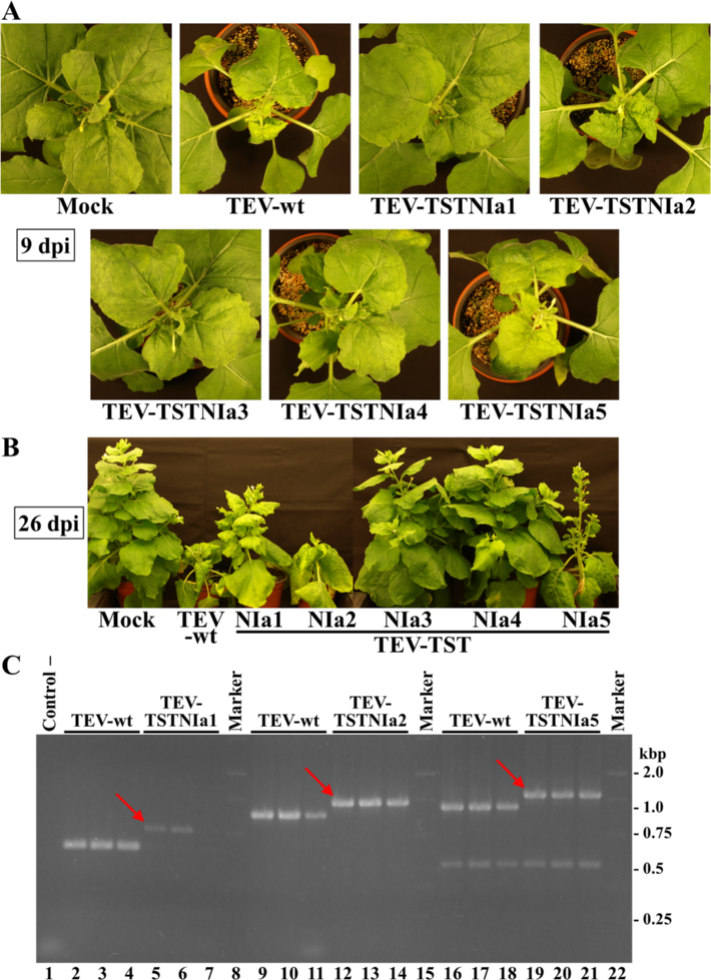
Infectivity of TEV recombinant clones with tagged NIa. **a** and **b** *N. benthamiana* plants non-inoculated (mock) and agroinoculated with wild-type TEV (TEV-wt) and TEV clones with TST-NIa at five different positions (TEV-TSTNIa1 to TEV-TSTNIa5). Pictures of representative plants were taken at 9 (**a**) or 26 (**b**) days postinoculation (dpi). **c** RT-PCR analysis of the viral progenies from plants infected with TEV-wt (lanes 2 to 4, 9 to 11 and 16 to 18), TEV-TSTNIa1 (lanes 5 to 7), TEV-TSTNIa2 (lanes 12 to 14) and TEV-TSTNIa5 (lanes 19 to 21). The control RT-PCR reaction (lane 1) contained all primers and no template. Lanes 8, 15 and 22, DNA marker ladder with sizes on the right side in kbp. Red arrows point the PCR products delayed as a consequence of insertion of the TST cDNA in the viral progenies

*Agrobacterium tumefaciens* was transformed with the binary plasmids bearing the recombinant TEV clones and cultures of this bacterium were used to agroinoculate *Nicotiana benthamiana* Domin plants. Nine days postinoculation (dpi), in addition to plants inoculated with wild-type TEV, all plants inoculated with TEV-TSTNIa2 and TEV-TSTNIa5 showed the typical symptoms of TEV infection. Half of the plants inoculated with TEV-TSTNIa1 also showed symptoms that were milder. None of the plants inoculated with TEV-TSTNIa3 and TEV-TSTNIa4 showed symptoms (Fig. 2a). Twenty-six dpi, plants inoculated with TEV-TSTNIa3 and TEV-TSTNIa4 remained undistinguishable from mock-inoculated plants (Fig. 2b). Reverse transcription (RT)-PCR analysis of inoculated plants confirmed that systemic tissues of non-symptomatic plants were free of the virus and systemic tissues of symptomatic plants contained TEV. Comparison of the electrophoretic mobility of a series of PCR products from plants infected with wild-type TEV and TEV-TSTNIa1, TEV-TSTNIa2 and TEV-TSTNIa5 at 9 dpi showed that viral progeny of these three recombinant viruses maintained the tag (Fig. 2c). The intensity of the bands also suggested that viral loads in the case of TEV-TSTNIa2 and TEV-TSTNIa5 were high and comparable to wild-type TEV, whereas TEV-TSTNIa1 load was lower (Fig. 2c, compare lanes 5 to 7 with lanes 2 to 4, 9 to 14 and 16 to 21). These results indicated that insertion of TST in both the selected carboxy-terminal positions of VPg and NIaPro does not strongly affect viral infectivity and accumulation. Hence, TEV-TSTNIa2 and TEV-TSTNIa5, containing the TST at the VPg and NIaPro domains respectively, were selected for further analysis.

### Identification of host proteins forming complex with TEV NIa in infected tissues

We mechanically inoculated three batches of *Arabidopsis thaliana* L. (ecotype L*er*-0) plants with extracts from *N. benthamiana* tissues infected with wild-type TEV, TEV-TSTNIa2 and TEV-TSTNIa5. Aerial tissues from plants infected with each virus were harvested 14 dpi and pooled. All plants showed symptoms of infection at this point. Western blot analysis of aliquots from the collected tissues using an anti-TST monoclonal antibody confirmed accumulation of TST-NIa and TST-VPg in plants infected with TEV-TSTNIa2, and TST-NIa and TST-NIaPro in plants infected with TEV-TSTNIa5. Bands corresponding to NIa products resulting from cleavage at the NIaPro carboxy-proximal autoproteolytic site [17, 18], were also observed. No signal was detected in non-inoculated controls and plants infected with wild-type TEV. Protein complexes containing TST-NIa-derived protein products were purified from the three batches of infected tissues by affinity chromatography in native conditions. Proteins from purified complexes were separated by denaturing polyacrylamide gel electrophoresis (PAGE) and digested in gel with trypsin. Peptides were then eluted from the gel and analyzed by liquid chromatography and tandem mass spectrometry to identify the proteins present in the purified complexes.

Additional file 2 lists all hits resulting from proteomic analysis of complexes purified from tissues infected with wild-type TEV, TEV-TSTNIa2 (VPg tagged) and TEV-TSTNIa5 (NIaPro tagged). Next, hits were classified according to the exponentially modified protein abundance index (emPAI) [21]. For each hit arising from complexes purified from the tissues infected with TEV-TSTNIa2 and TEV-TSTNIa5, we calculated the difference in emPAI (ΔemPAI) with respect to the tissues infected with wild-type TEV. Hits were considered spurious if they matched any of the following conditions: (i) proteins were identified in very low abundance (emPAI lower than a given threshold value), despite not being detected in wild-type TEV infected control tissues; and (ii) they were identified in relatively high abundance but also detected in wild-type TEV infected control tissues in a way that ΔemPAI is also under a given threshold. Remaining hits were computationally filtered and assigned to single genes of the *A. thaliana* genome (Additional file 3).

### TEV NIa-host interaction network

Our experimental approach resulted in a total of 232 different host proteins considered to be involved in protein complexes in which TEV NIa processing products take also part, according to a ΔemPAI threshold criterion. More in detail, 156 and 143 host proteins were identified when the affinity tag was inserted close to the carboxyl terminus of the VPg and NIaPro domains, respectively; 67 proteins were common to both lists. This common set of proteins most probably results from complexes in which the full-length NIa is involved, although they may also indicate redundancy in the mode of action of VPg and NIaPro. Hereafter, the 232 host proteins were classified as specific VPg targets (89 proteins), specific NIaPro targets (76 proteins) and NIa targets (67 proteins). In all cases, it is important to stress out that targets may be direct or indirect. Overall, these results suggest that, similarly to what occurs with RNA viruses infecting animal cells [3, 4], proteins from plant RNA viruses also target many host elements during infection.

**Fig. 3:**
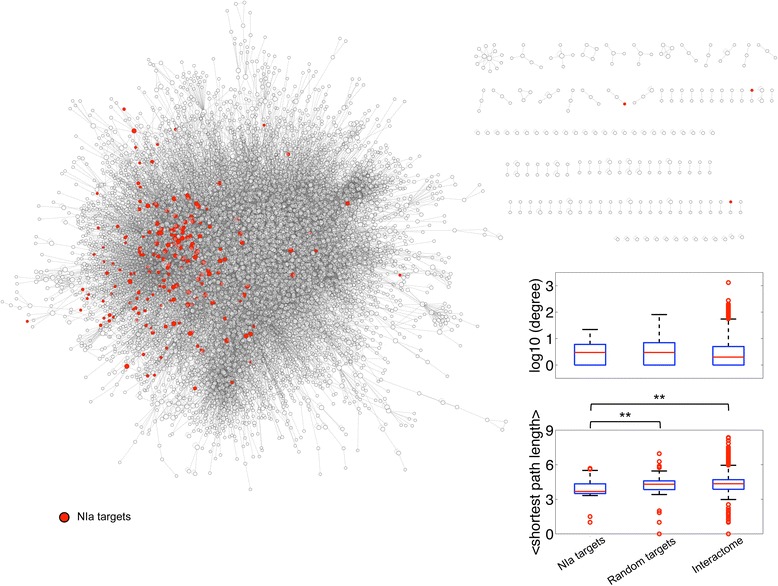
Contextualization of the TEV NIa host targets on the experimental *A. thaliana* interactome. Illustration of the global interactome together with a boxplot of the global topological properties (degree and average shortest path length) of the NIa targets. Note that these properties were calculated according to the global interactome and not to a particular subnetwok. No significant differences were found between distributions of degree. By contrast, differences were significant between distributions of average shortest path length (Mann–Whitney *U*-test, *P* < 0.0001). As controls, the distributions of a representative random sample and the whole interactome are shown

Next, we contextualized the identified targets in the experimental *A. thaliana* protein-protein interactome (Fig. 3 and Additional file 4). NIa targets indicated in Fig. 3 represent either direct or indirect interactions. Of the 232 host proteins, 49 were not covered in the *A. thaliana* interactome, which only contains about 8,000 proteins (Additional file 4). We found that NIa targets are not more connected with other host proteins than expected by chance (Fig. 3). It has been shown, however, that virus targets tend to be hubs in the host interactome [4, 22]. According to our data, this is not the case of TEV NIa. Instead, our network-based analysis revealed that the detected targets are in a position that allows them to connect with other host proteins throughout short paths (much shorter than expected by chance; Fig. 3). That is, NIa targets connect on average in 3.68 steps with any other protein of the interactome, whilst randomly picked proteins do in 4.30 steps (Mann–Whitney *U*-test, *P* < 0.0001). This indicates that local perturbations by the virus in the host interactome (e.g., inhibition of the function of a given host factor by protein-protein interaction) could reach a global outcome very quickly [23], leading to significant changes in the physiology of the cell and then in the whole organism (e.g., observable symptoms in the plant upon infection). No significant differences were found between the three categories: VPg, NIaPro and full-length NIa targets (Additional file 5). Even though the *A. thaliana* interactome is incomplete, a limitation that has not prevented the distillation of the mechanisms of important diseases in humans [24], our results suggest that NIa targets are elements that can efficiently transmit information through the host interactome [25].

In the *A. thaliana* interactome, the most connected elements specifically targeted by VPg are the G-box regulating factor 6 (GRF6, a 14-3-3 protein λ isoform) with degree 18, and a mitochondrial ATP synthase (δ subunit) with degree 15. The former protein has been shown to participate in plant defense [26]. Different proteins from animal RNA viruses, like *Hepatitis C virus* core protein, have also been shown to bind to 14-3-3 proteins [27], inducing disease by triggering certain kinase cascades, whilst the most connected proteins specifically targeted by NIaPro are the plasma membrane aquaporin PIP2;7 with degree 16, and the actin 7 with degree 11. In particular, PIP2;7 has been shown to mediate response to stress [28] and is highly clustered in the network. The most connected proteins targeted by NIa (targets common to VPg and NIaPro) are the heat shock cognate protein 70–1 (Hsp70-1) with degree 22, and the photosystem protein LHCA3 with degree 16. Hsp70 proteins are ubiquitous molecular chaperones that act in polypeptide folding, refolding of misfolded or aggregated proteins, translocation across membranes, protein complex assembly and protein degradation [29, 30]. They play central roles in the formation of membrane-bound replication complexes in many plant viruses, including potyviruses [31, 32]. Of relevance, our data suggest that full-length NIa (or both domains separately) binds to a protein complex that includes the host pathogenesis-related protein PR-5. This interaction may indicate a viral strategy to interfere with the host defense mechanisms, as systemic acquired resistance against pathogens is associated with the accumulation of salicylic acid and PR proteins [33]. In addition, we identified some host proteins previously shown to interact with potyviral NIa or NIa domains, like the poly(A)-binding proteins PABP4 and PABP8 [34–36], the translation elongation factor EF1A [37], and a methionine sulfoxide reductase [38]. However, we did not detect other interactors previously shown using different techniques for TEV or other potyviruses, like the translation initiation factors eIF4E or eIF(iso)4E [12, 39–41], the helicase RH8 [42], the fibrillarin [43], the elongin C [44], and the silencing-related protein SGS3 [45]. In any case, note that some of these interactions may be species specific. For instance, we neither detected interaction with PHD-finger proteins OBE1 and OBE2 (initially reported for *Pea seed borne mosaic virus*), but TEV VPg failed to interact with these proteins in a yeast to hybrid system [46]. A recent review summarizes most host proteins that have currently shown to interact with potyviral proteins [47].

**Fig. 4:**
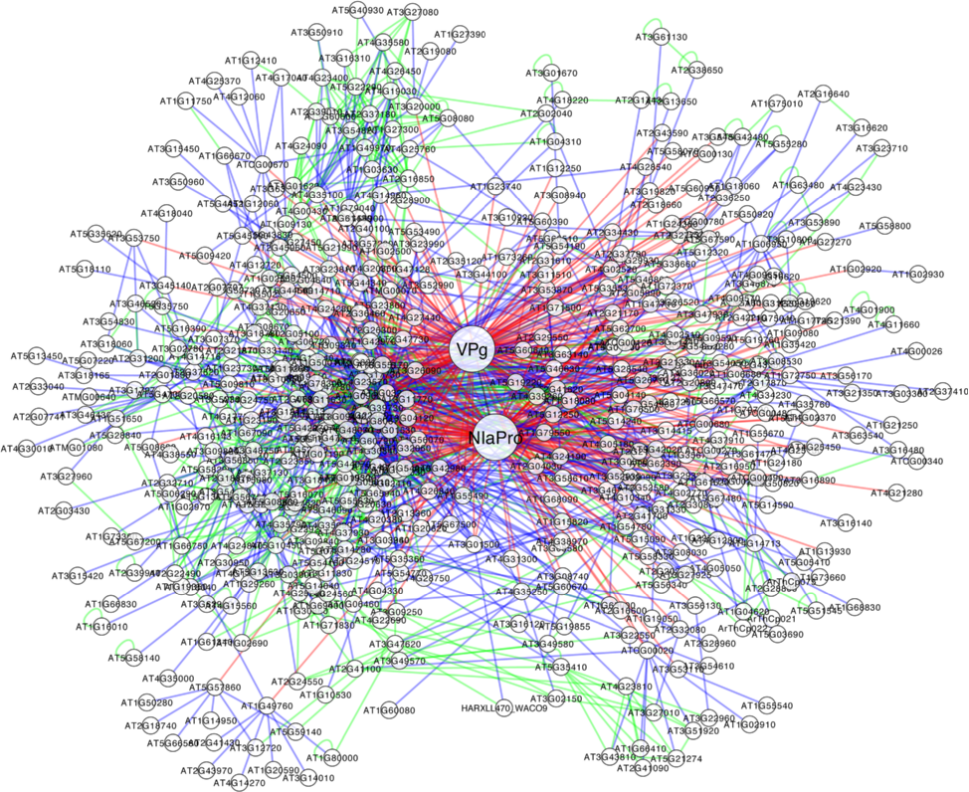
Protein-protein interaction network between the two domains of TEV NIa (VPg and NIaPro) and *A. thaliana* proteins during infection. Interactions between viral and host proteins (VPg, NIaPro and NIa targets) are shown in red, between the virus targets and their direct partners in the host in blue, and between host proteins not being targeted by NIa in green. Map was constructed with Cytoscape

Next, we constructed a NIa-host protein interaction network (Fig. 4). This network also includes *A. thaliana* proteins that have been well established to interact (Additional file 4) with those experimentally identified in our work. We obtained a network containing 516 host proteins connected to the two domains of NIa. Note that 49 proteins are not connected to other host proteins in our network. We calculated an average connectivity of 5.24, a characteristic path length of 3.05, and an average clustering coefficient of 0.16. The resulting degree distribution is scale-free with an exponent of −1.67 (Additional file 6). We anticipate that this network will serve to better understand the mode of action of potyviruses, and then serve to develop effective strategies for crop protection or infection limitation.

### Functional analysis of the host proteins identified in complexes with TEV NIa

**Fig. 5:**
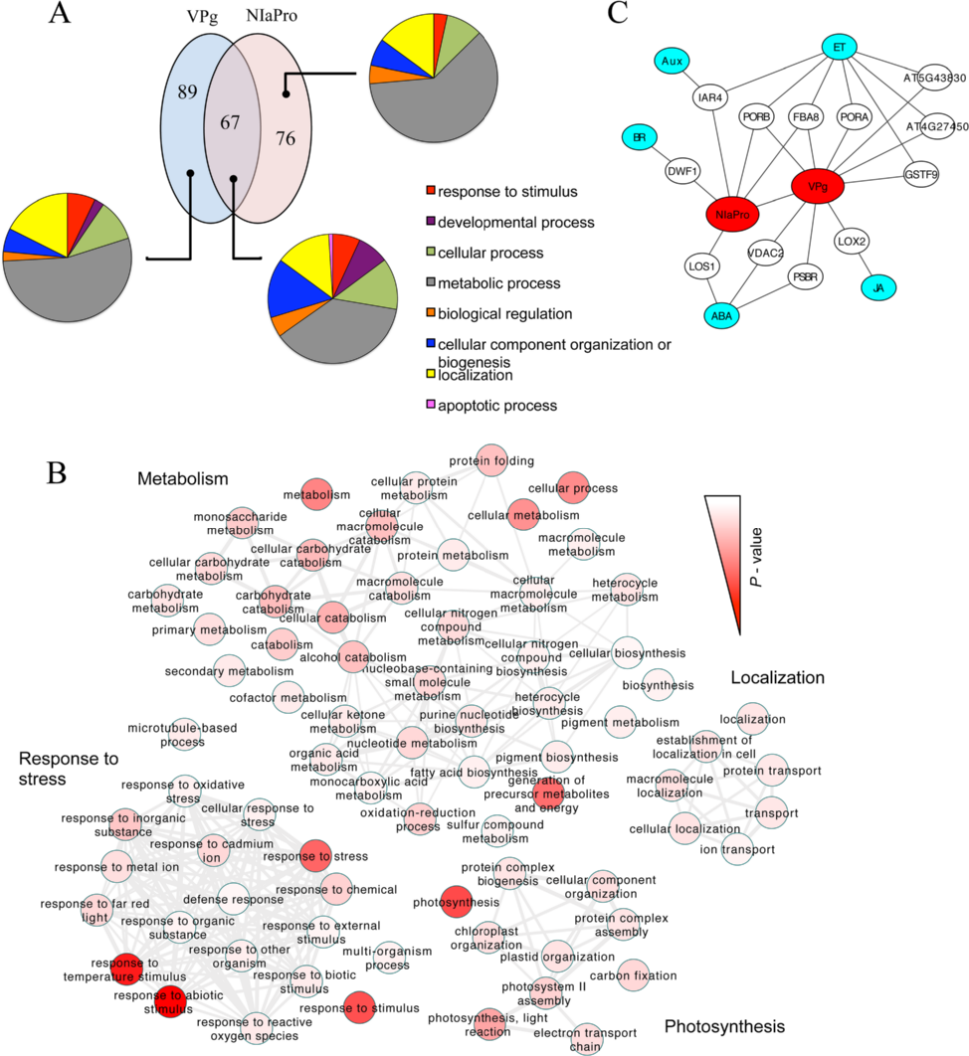
TEV NIa-host functional networks. **a** Venn diagrams showing the number of host proteins targeted by the VPg and NIaPro domains of TEV NIa during infection, together with a functional analysis of the global categories over-represented. **b** Map of the different biological functions (GO terms) over-represented within the complete list of targeted host proteins (VPg, NIaPro and common). Representation obtained with REVIGO. Red intensity corresponds to the *P*-value. **c** Network representing the relationship between NIa-targeted host proteins and phytohormones. This network was obtained taking advantage of the Arabidopsis Hormone Database

From our refined lists of host proteins targeted by NIa products during infection (Additional file 3), we identified the global functional categories of VPg and NIaPro specific targets, as well as common targets, using the Panther tool [48] (Fig. 5a). Regarding plant physiology, we observed that specific targets impact more metabolism, whilst those in common do development and organization. We found that common targets are significantly enriched (adjusted *P* < 0.05) in genes coding for the response to biotic stimulus (GO:0009607), whilst specific targets of VPg and NIaPro are not. In particular, the specific targets of VPg are less involved in response to stimulus with respect to the 67 shared elements (Fisher exact test for GO:0050896, *P* < 0.05). Moreover, we obtained a functional network of over-represented gene ontology (GO) terms for the complete list of NIa targets (232 elements) (Fig. 5b and Additional file 3), using the agriGO tool [49]. In this network, functional categories are shown to be related to each other, with proteins belonging to different categories. Over-represented GO terms were mainly grouped in response to stress (biotic and abiotic), metabolism, photosynthesis and localization. We found that both VPg and NIaPro targets are similarly engaged in these main groups (Fisher exact tests for GO:0050896, GO:0008152, GO:0015979, and GO:0051179, *P* > 0.05), perhaps because almost 50 % of targets are shared. We also observed that, in general, NIa targets more proteins related to abiotic stress than biotic stress (Fisher exact test for GO:0009628 vs. GO:0009607, *P* < 0.05).

Finally, by taking advantage of known host proteins involved in biosynthesis, signal transduction and response of phytohormones [50], we delineated a new functional map of NIa during infection (Fig. 5c). Ethylene, which plays a crucial role in mounting defense responses, particularly during pathogen infections [51], was shown to interact with the majority of the identified proteins. Auxins and brassinosteroids were linked to NIaPro, which may suggest an effect on plant development, whilst jasmonic acid, which exhibits a big cross-talk with salicylic acid, was associated to VPg.

### Remarks about the experimental approach

Several issues should be taken into consideration about these interaction networks. First, our lists of host NIa-targeted proteins come from an AP-MS experimental approach. Some of the identified proteins may still result from spurious interactions in vivo or during the purification process and be functionally irrelevant. At the same time, some true interactors may have been missed due to low abundance, transient binding or inappropriate experimental conditions. Second, our experimental approach searches for host proteins bound to multiprotein complexes to which the tagged viral proteins also bind. This way, reported host interactors may not necessarily bind directly to the viral proteins. Certainly, the use of a thorough pipeline (e.g., AP-MS combined with yeast two-hybrid or bimolecular fluorescence complementation screenings) will help to find the direct interactors and to decipher the supramolecular organization of protein complexes [6]. Third, our experimental results derive from analyses of pooled plants and tissues at 2-week after inoculation. Ours is probably a snapshot at a late stage of infection. However, host-virus interactions are dynamic, establishing and releasing connections in each cell as the infection progresses. Thus, some known interactions between NIa domains and host factors may not exist or be underrepresented at our sampling time. Further AP-MS analyses at different times post-inoculation will be required to uncover the dynamics of host-virus interaction network along the infectious cycle.

## Conclusions

An affinity tag, such as TST, can be inserted between the codon positions -9/-8 in the VPg and NIaPro domains of TEV NIa without strongly affecting virus viability. Remarkably, the TST tag inserted in either of these two positions is stable in the viral progenies, allowing affinity purification of protein complexes in which NIa processing products are involved. TEV NIa targets many host proteins during infection. Our AP-MS approach applied to *A. thaliana* tissues infected with TST-tagged TEV clones detected 89 and 76 host proteins specifically involved in complexes with the VPg and NIaPro domains of NIa, respectively, and 67 common proteins, most probably full-length NIa targets. Combining these experimental data with currently known information about the *A. thaliana* interactome, we constructed a NIa-host protein-protein interaction network containing 516 host proteins connected to the two domains of NIa. This network has an average connectivity of 5.24, a characteristic path length of 3.05, and an average clustering coefficient of 0.16. The network also highlights that the most connected host elements are the G-box regulating factor 6 (degree 18, specifically targeted by VPg) and the heat shock cognate protein 70–1 (degree 22, commonly targeted by VPg and NIaPro or by full-length NIa). Further analyses of the host proteins targeted by NIa processing products allowed constructing additional NIa-host interaction networks. These networks show that NIa-targeted proteins are mainly involved in response to stress (biotic and abiotic), metabolism, photosynthesis and localization, and that ethylene is the phytohormone most connected with the NIa-targeted host proteins. Finally, a look at the position of the detected targets in the global *A. thaliana* interactome reveals that NIa targets are not more connected with other host proteins than expected by chance, but are in a position that allows them to connect with other host proteins in shorter paths, which may suggest a strategy to efficiently transmit information.

## Methods

### Plasmid construction

Binary plasmid pGTEVat contains an infectious TEV clone (sequence variant DQ986288, including mutations G273A, A1119G and C6037T) under the control of CaMV 35S promoter and terminator. Mutations G273A and A1119G are synonymous and apparently neutral, and mutation C6037T replaces an amino acid in VPg (L1965F) increasing infectivity and viral load in *A. thaliana* ecotype L*er*-0 [52]. pGTEVat was constructed from pGTEVa [53], by inserting the C6037T mutation by PCR using the high-fidelity Phusion DNA polymerase (Thermo Scientific). TEV sequence in pGTEVat was modified by PCR at positions 5700–5701, 6231–6232, 6264–6265, 6888–6889, 6957–6958 using the pairs of primers I (5′-CCGCggtctcC**CGCT**CTTCTTCCCTTGGAAATAGAC-3′) and II (5′-GGCGggtctcG**CGGA***GGGAAAAAG*AATCAGAAGCAC-3′), III (5′-CCGCggtctcC**CGCT**TGGTGGCAATTGATCATAAG-3′) and IV (5′-GGCGggtctcG**CGGA**AAGAGTGAGGACTTGACGTTTG-3′), V (5′-CCGCggtctcC**CGCT**GCTTTCTCCTTCAAACGTCAAG-3′) and VI (5′-GGCGggtctcG**CGGA***GGAGAGAGC*TTGTTTAAGGG-3′), VII (5′-CCGCggtctcC**CGCT**CCACAATACTGAGTCAGCATTTA-3′) and VIII (5′-GGCGggtctcG**CGGA**GGGGGCCATAAAGTTTTCATG-3′), and IX (5′-CCGCggtctcC**CGCT**GAGTTGAGTCGCTTCCTTAAC-3′) and X (5′-GGCGggtctcG**CGGA**ATGAGTGAATTGGTGTACTC-3′), respectively. These primers inserted a small polylinker with two inverted recognition sites for Eco31I, a type-IIS restriction enzyme. Eco31I recognition and cleavage sequences are in lower font and bold, respectively, in above primers. Primers II and VI also inserted three-codon duplications (nucleotides in italics in above sequences). Plasmid pUBSt contains the TST cDNA flanked by two Eco31I restriction sites (5′- ggtctcG**AGCG**CA***TGGAGTCATCCTCAATTCGAGAAA***GGTGGAGGTTCTGGCGGTGGATCGGGAGGTTCAGCG***TGGAGCCACCCGCAGTTCGAAAAA***TC**CGGA**Ggagacc-3′, nucleotides corresponding to Strep-Tactin binding domains in bold italics; the remaining nucleotides correspond to the TST spacer regions; Eco31I recognition and cleavage sequences are in lower font and bold, respectively) inserted in the SmaI site of pUC18 (GenBank accession number L08752). By digestion with Eco31I (Thermo Scientific) and ligation with T4 DNA ligase (Thermo Scientific), TST cDNA was transferred from pUBSt to the five plasmids derived from pGTEVat. The sequences of the resulting plasmids (pGTEVat-TSTNIa1 to 5) were confirmed by standard DNA sequencing techniques.

### Plant inoculation

*A. tumefaciens* C58C1 harboring the helper plasmid pCLEAN-S48 [54] was transformed with plasmids pGTEVat-TSTNIa1 to 5. Batches of six 4.5-week-old *N. benthamiana* plants were agroinoculated with cultures of transformed *A. tumefaciens*. Briefly, bacteria were adjusted to an optical density of 0.5 at 600 nm in 10 mM MES-NaOH, pH 5.6, 10 mM MgCl_2_, virulence genes induced with 150 μM acetosyringone for 2 h at 28 °C, and infiltrated underneath plant leaves using a syringe without needle [55].

Three batches of 48 *A. thaliana* plants were mechanically inoculated with extracts from *N. benthamiana* tissues infected with wild-type TEV, TEV-TSTNIa2 and TEV-TSTNIa5, using a cotton swab in the presence of Carborundum [53]. Extracts were obtained by grinding tissue in the presence of 20 volumes of inoculation buffer (50 mM potassium phosphate, pH 8.0, 1 % polyvinylpyrrolidone 10, 1 % polyethylene glycol 6000, and 10 mM 2-mercaptoethanol).

### Analysis of viral progeny

From 100-aliquots of systemic tissue of three inoculated plants for each virus, RNA was purified using silica gel spin columns (Zymo Research) [56]. For TEV diagnosis, RNA was subjected to reverse transcription using M-MuLV reverse transcriptase (Revertaid, Thermo Scientific) using primer 5′-CTCGCACTACATAGGAGAATTAGAC-3′. Products of reverse transcription were amplified by PCR with *Thermus thermophilus* DNA polymerase (Biotools) using primers 5′-AGTGGCACTGTGGGTGCTGGTGTTG-3′ and 5′-CTGGCGGACCCCTAATAG-3′ and revealed by gel electrophoresis (1 % agarose) and ethidium bromide staining.

To analyze the presence of the TST cDNA in the viral progenies, all reverse transcription reactions were primed with 5′-AGGAACGCCTCTCTATTAAGTCGAC-3′. PCR amplifications were performed with primers 5′-TCAGATAGCGAAGTGGCTAAGCATC-3′ and 5′- TGACCTGTCAATGGATCCACAAACC-3′ (TEV-wt and TEV-TSTNIa1), 5′- TTAGGTTTGTGGATCCATTGACAGG-3′ and 5′- ATGGTGGGAAATCCTTAGGCATGCG-3′ (TEV-wt and TEV-TSTNIa2) and 5′- TTATTCGCATGCCTAAGGATTTCCC-3′ and 5′- AGGAACGCCTCTCTATTAAGTCGAC-3′ (TEV-wt and TEV-TSTNIa5). Amplification products were separated by 1 % agarose electrophoresis and stained with ethidium bromide.

### Protein purification

Protein complexes containing TST-NIa-derived protein products were purified from 15 g of each of the three batches of *A. thaliana* infected tissues by affinity chromatography in native conditions using a 1-ml Strep-Tactin Superflow column (IBA) as previously described [20]. Briefly, tissues were ground in a mortar in the presence of liquid N_2_, homogenized with three volumes (45 ml) of extraction buffer (100 mM Tris–HCl, pH 8.0, 150 mM KCl, 10 mM MgCl_2_, 10 mM dithiothreitol − DTT−, 1 mM EDTA, 1 % Nonidet P-40) including a cocktail of protease inhibitors (Complete, Roche Life Science), and clarified twice by centrifugation at 4 °C, at 12,000 × g for 15 and at 95,000 × g for 30 min. Protein purification was performed with an ÄKTA Prime Plus liquid chromatography system (GE Healthcare) operated at 4 °C at a flow rate of 1 ml/min. After column equilibration with 10 ml of extraction buffer, the column was loaded with the clarified extract and washed with 20 ml of extraction buffer. Bound protein complexes were eluted with 20 ml of extraction buffer containing 10 mM D-desthiobiotin. Fractions (0.5 ml) were analyzed by Western blotting with an anti-TST antibody (StrepMAB Classic-HRP, IBA). Selected fractions were pooled and proteins precipitated with 4 volumes of 12.5 % trichloroacetic acid and 10 mM DTT in acetone. The same process was followed with the corresponding fractions eluted in the control purification process from tissues infected by TEV-wt.

### Protein identification

Protein preparations were separated by PAGE in the presence of sodium dodecyl sulfate (SDS; 12.5 % polyacrylamide, 0.05 % SDS). The gel was stained with Coomassie blue and whole lanes corresponding to each sample were excised and cut in pieces. Proteins were subjected to in-gel digestion with sequencing-grade trypsin (Promega) [57]. Peptides were eluted from the gel pieces and analyzed by liquid chromatography and tandem mass spectrometry (LC-MS/MS). Samples (5 μl) were loaded onto a trap column (NanoLC column, 3 μ C18-CL, 100 μm × 15 cm; Nikkyo) and desalted with 0.1 % trifluoroacetic acid (TFA) at 3 μl/min during 5 min. Peptides were then loaded onto an analytical column (LC column, 3 μ C18-CL, 75 μm × 12 cm; Nikkyo) equilibrated in 5 % acetonitrile (ACN) and 0.1 % formic acid (FA). Elution was carried out with a linear gradient of 5 to 40 % B in 50 min (A: 0.1 % FA; B: ACN, 0.1 % FA) at a flow rate of 300 nl/min. Peptides were analyzed in a mass spectrometer nanoESI qQTOF (5600 TripleTOF, ABSCIEX). The tripleTOF was operated in information-dependent acquisition mode, in which a 0.25-s TOF MS scan from 350–1250 m/z, was performed, followed by 0.05-s product ion scans from 100–1500 m/z on the 50 most intense 2–5 charged ions. For abundant bands the analysis was done in the same way with a 30 min gradient. Protein identification was performed using ProteinPilot v4.5 (ABSciex) and Mascot v2.2 (Matrix Science) search engines. ProteinPilot default parameters were used to generate peak list directly from 5600 TripleTof whiff files. The Paragon algorithm of ProteinPilot was used to search NCBI protein database (22470027 proteins searched) with the following parameters: trypsin specificity, cys-alkylation, no taxonomy restriction, and the search effort set to thorough. To avoid using the same spectral evidence in more than one protein, the identified proteins are grouped based on MS/MS spectra by the ProteinPilot Progroup algorithm. Thus, proteins sharing MS/MS spectra are grouped, regardless of the peptide sequence assigned. The protein within each group that can explain more spectral data with confidence is shown as the primary protein of the group. Only the proteins of the group for which there is individual evidence (unique peptides with enough confidence) are also listed, usually toward the end of the protein list.

### Computational analysis

A bioinformatic mapping to single genes of *A. thaliana* genome was performed with the final lists of protein hits detected by Mascot (with GenInfo Identifier − GI−), obtaining the arabidopsis information resource (TAIR) identifiers. Repeated elements were removed. When the detected protein was not from *A. thaliana*, a sequence alignment was done with BLAST [58] to obtain the corresponding ortholog in *A. thaliana*. Each hit had associated an exponentially modified protein abundance index (emPAI) [21]. Only hits with a differential emPAI of ΔemPAI ≥ 0.1 were selected (tissues infected with TEV-TSTNIa2 and TEV-TSTNIa5 versus tissues infected with wild-type TEV). Note that 0.1 approximately corresponds to the median of the distributions in ΔemPAI for VPg and NIaPro hits. An emPAI = 0 was assumed for elements not appearing from AP-MS of tissues infected with wild-type TEV.

With the filtered list of *A. thaliana* gene identifiers (NIa targets), protein-protein interaction network was constructed with Cytoscape [59] using the experimental *A. thaliana* interactome. For that, host proteins with direct interaction with the detected NIa targets were selected. Each protein targeted by NIa was contextualized in the interactome to analyze in a quantitative manner its position. The global topological properties of the resulting network were also calculated. Random lists of proteins were also generated to obtain control distributions of topological properties by picking randomly a given number of genes from the *A. thaliana* genome (about 180 in this case). The *A. thaliana* interactome was constructed thanks to a high-throughput identification of binary protein-protein interactions in yeast [60, 61] and also by accounting for all known interactions in the BIOGRID database [62]. The interactome covers about 8,000 proteins and has about 22,000 non-redundant interactions, all of them with experimental evidence. The interactome used in this work is provided in the Additional file 4.

A global functional enrichment of the proteins specifically targeted by VPg, by NIaPro, or by both (common targets) was performed using Panther [48]. Functional analyses using agriGO [49] were also performed to identify the biological processes over-represented within the complete list of NIa targets (VPg, NIaPro and NIa). The statistical significance, of the list with respect to the complete plant genome (TAIR9), was evaluated by a Fisher exact test (2 × 2 contingency tables) with a correction for multiple testing using the Benjamini-Hochberg FDR procedure (adjusted *P* < 0.05), only considering GO terms with five or more mapping entries. With those identified functional categories, a functional network was constructed and using REVIGO [63], where functions sharing elements were connected. Sublists of genes were also analyzed. A network representing the relationship between VPg and NIaPro-targeted host proteins and phytohormones was also constructed taking advantage of the Arabidopsis Hormone Database [50].

## Additional files

Additional file 1:
**cDNA sequences of wild-type NIa (from nucleotide 5692 to 6981 of GenBank accession DQ986288) and the five tagged variants TSTNIa1 to 5.** Twin-Strep-tag (TST) sequence is on yellow background; codons corresponding to amino acids binding Strep-Tactin and to spacers in blue and gray, respectively. Positions corresponding to the different NIaPro autoproteolytic sites are on blue background. Mutation C6037T that increases viral load and infectivity in *A. thaliana* Ler-0 is in red. In TSTNIa1 and TSTNIa3 the three codons in front of TST are duplicated (italics) after the tag, but include a silent mutation (in red) to avoid homologous repetitions. (PDF 66 kb) Additional file 2:
**Protein hits obtained by mass spectrometry analysis of protein complexes purified by affinity chromatography from** ***A. thaliana*** **tissues infected with wild-type** **TEV (negative control)**, **TEV-NIaTST2 (VPg tagged) and TEV-NIaTST5 (NIaPro tagged).** (XLSX 356 kb) Additional file 3:
**Refined lists of** ***A. thaliana*** **single genes targeted by TEV VPg and NIaPro domains of NIa during infection, respectively.** Note that 67 elements are common to both lists (full-length NIa targets). The file also contains the list of the gene ontology (GO) terms significantly over-represented in the global list of NIa targets. (XLSX 95 kb) Additional file 4:
**TEV NIa (VPg and NIaPro)-host interaction network constructed in this work.** This file also contains the experimental *A. thaliana* protein interactome. (XLSX 393 kb) Additional file 5:
**Boxplot of the global topological properties (degree and average shortest path length) of the NIa targets covered in the experimental** ***A. thaliana*** **interactome.** Note that these properties were calculated according to the global interactome and not to a particular subnetwok. No significant differences were found between distributions, except for the comparison in degree between specific VPg targets and those in common (Mann–Whitney *U*-test, *P* = 0.03). (PDF 21 kb) Additional file 6:
**Degree distribution of the NIa network showing a scale-free trend.** The three outliers with higher degree were not considered in the regression. (PDF 38 kb)

## Abbreviations

NIa: nuclear inclusion a
AP-MS: affinity purification coupled to mass spectrometry
VPg: viral protein genome-linked
NIaPro: nuclear inclusion a protease domain
eIF4E: eukaryotic translation initiation factor 4E
NIb: nuclear inclusion b
TEV: tobacco etch virus
CaMV: cauliflower mosaic virus
TST: Twin-Strep-tag
dpi: days postinoculation
RT: reverse transcription
emPAI: exponentially modified protein abundance index
GO: gene ontology
