# Supplementary material for: Interaction network of tobacco etch potyvirus NIa protein with the host proteome during infection
Source: BMC Genomics. 2016 Feb 1;17:87. doi: 10.1186/s12864-016-2394-y (PMC4735970; doi:10.1186/s12864-016-2394-y)
Supplement: Additional file 1: — cDNA sequences of wild-type NIa (from nucleotide 5692 to 6981 of GenBank accession DQ986288) and the five tagged variants TSTNIa1 to 5. Twin-Strep-tag (TST) sequence is on yellow background; codons corresponding to amino acids binding Strep-Tactin and to spacers in blue and gray, respectively. Positions corresponding to the different NIaPro autoproteolytic sites are on blue background. Mutation C6037T that increases viral load and infectivity in A. thaliana Ler-0 is in red. In TSTNIa1 and TSTNIa3 the three codons in front of TST are duplicated (italics) after the tag, but include a silent mutation (in red) to avoid homologous repetitions. (PDF 66 kb) [file 12864_2016_2394_MOESM1_ESM.pdf]

Additional file 1: cDNA sequences of wild-type NIa (from nucleotide 5692 to 6981 of GenBank accession DQ986288) and the five tagged variants TSTNIa1 to 5. Twin-Strep-tag (TST) sequence is on yellow background; codons corresponding to amino acids binding Strep-Tactin and to spacers in blue and gray, respectively. Positions corresponding to the different NIaPro autoproteolytic sites are on blue background. Mutation C6037T that increases viral load and infectivity in *A. thaliana* Ler-0 is in red. In TSTNIa1 and TSTNIa3 the three codons in front of TST are duplicated (italics) after the tag, but include a silent mutation (in red) to avoid homologous repetitions.

>NIa
GGGAAGAAGAATCAGAAGCACAAGCTTAAGATGAGAGAGGCGCGTGGGGCTAGAGGGCAATATGAGGTTGCAGCG
GAGCCAGAGGCGCTAGAACATTACTTTGGAAGCGCATATAATAACAAAGGAAAGCGCAAGGGCACCACGAGAGGA
ATGGGTGCAAAGTCTCGGAAATTCATAAACATGTATGGGTTTGATCCAACTGATTTTTCATACATTAGGTTTGTG
GATCCATTGACAGGTCACACTATTGATGAGTCCACAAACGCACCTATTGATTTAGTGCAGCATGAGTTTGGAAAG
GTTAGAACACGCATGTTAATTGACGATGAGATAGAGCCTCAAAGTTTTAGCACCCACACCACAATCCATGCTTAT
TTGGTGAATAGTGGCACGAAGAAAGTTCTTAAGGTTGATTTAACACCACACTCGTCGCTACGTGCGAGTGAGAAA
TCAACAGCAATAATGGGATTTCCTGAAAGGGAGAATGAATTGCGTCAAACCGGCATGGCAGTGCCAGTGGCTTAT
GATCAATTGCCACCAAAGAGTGAGGACTTGACGTTTGAAGGAGAAAGCTTGTTTAAGGGACCACGTGATTACAAC
CCGATATCGAGCACCATTTGTCACTTGACGAATGAATCTGATGGGCACACAACATCGTTGTATGGTATTGGATTT
GGTCCCTTCATCATTACAAACAAGCACTTGTTTAGAAGAAATAATGGAACACTGTTGGTCCAATCACTACATGGT
GTATTCAAGGTCAAGAACACCACGACTTTGCAACAACACCTCATTGATGGGAGGGACATGATAATTATTCGCATG
CCTAAGGATTTCCCACCATTTCCTCAAAAGCTGAAATTTAGAGAGCCACAAAGGGAAGAGCGCATATGTCTTGTG
ACAACCAACTTCCAAACTAAGAGCATGTCTAGCATGGTGTCAGACACTAGTTGCACATTCCCTTCATCTGATGGC
ATATTCTGGAAGCATTGGATTCAAACCAAGGATGGGCAGTGTGGCAGTCCATTAGTATCAACTAGAGATGGGTTC
ATTGTTGGTATACACTCAGCATCGAATTTCACCAACACAAACAATTATTTCACAAGCGTGCCGAAAAACTTCATG
GAATTGTTGACAAATCAGGAGGCGCAGCAGTGGGTTAGTGGTTGGCGATTAAATGCTGACTCAGTATTGTGGGGG
GGCCATAAAGTTTTCATGAGCAAACCTGAAGAGCCTTTTCAGCCAGTTAAGGAAGCGACTCAACTCATGAGTGAA
TTGGTGTACTCGCAA

>TSTNIa1
GGGAAGAAGAGCGCATGGAGTCATCCTCAATTCGAGAAAGGTGGAGGTTCTGGCGGTGGATCGGGAGGTTCAGCG
TGGAGCCACCCGCAGTTCGAAAAATCCGGA*GGGAAAAAG*AATCAGAAGCACAAGCTTAAGATGAGAGAGGCGCGT
GGGGCTAGAGGGCAATATGAGGTTGCAGCGGAGCCAGAGGCGCTAGAACATTACTTTGGAAGCGCATATAATAAC
AAAGGAAAGCGCAAGGGCACCACGAGAGGAATGGGTGCAAAGTCTCGGAAATTCATAAACATGTATGGGTTTGAT
CCAACTGATTTTTCATACATTAGGTTTGTGGATCCATTGACAGGTCACACTATTGATGAGTCCACAAACGCACCT
ATTGATTTAGTGCAGCATGAGTTTGGAAAGGTTAGAACACGCATGTTAATTGACGATGAGATAGAGCCTCAAAGT
TTTAGCACCCACACCACAATCCATGCTTATTTGGTGAATAGTGGCACGAAGAAAGTTCTTAAGGTTGATTTAACA
CCACACTCGTCGCTACGTGCGAGTGAGAAATCAACAGCAATAATGGGATTTCCTGAAAGGGAGAATGAATTGCGT
CAAACCGGCATGGCAGTGCCAGTGGCTTATGATCAATTGCCACCAAAGAGTGAGGACTTGACGTTTGAAGGAGAA
AGCTTGTTTAAGGGACCACGTGATTACAACCCGATATCGAGCACCATTTGTCACTTGACGAATGAATCTGATGGG
CACACAACATCGTTGTATGGTATTGGATTTGGTCCCTTCATCATTACAAACAAGCACTTGTTTAGAAGAAATAAT
GGAACACTGTTGGTCCAATCACTACATGGTGTATTCAAGGTCAAGAACACCACGACTTTGCAACAACACCTCATT
GATGGGAGGGACATGATAATTATTCGCATGCCTAAGGATTTCCCACCATTTCCTCAAAAGCTGAAATTTAGAGAG
CCACAAAGGGAAGAGCGCATATGTCTTGTGACAACCAACTTCCAAACTAAGAGCATGTCTAGCATGGTGTCAGAC
ACTAGTTGCACATTCCCTTCATCTGATGGCATATTCTGGAAGCATTGGATTCAAACCAAGGATGGGCAGTGTGGC
AGTCCATTAGTATCAACTAGAGATGGGTTCATTGTTGGTATACACTCAGCATCGAATTTCACCAACACAAACAAT
TATTTCACAAGCGTGCCGAAAAACTTCATGGAATTGTTGACAAATCAGGAGGCGCAGCAGTGGGTTAGTGGTTGG
CGATTAAATGCTGACTCAGTATTGTGGGGGGGCCATAAAGTTTTCATGAGCAAACCTGAAGAGCCTTTTCAGCCA
GTTAAGGAAGCGACTCAACTCATGAGTGAATTGGTGTACTCGCAA

>TSTNIa2
GGGAAGAAGAATCAGAAGCACAAGCTTAAGATGAGAGAGGCGCGTGGGGCTAGAGGGCAATATGAGGTTGCAGCG
GAGCCAGAGGCGCTAGAACATTACTTTGGAAGCGCATATAATAACAAAGGAAAGCGCAAGGGCACCACGAGAGGA
ATGGGTGCAAAGTCTCGGAAATTCATAAACATGTATGGGTTTGATCCAACTGATTTTTCATACATTAGGTTTGTG
GATCCATTGACAGGTCACACTATTGATGAGTCCACAAACGCACCTATTGATTTAGTGCAGCATGAGTTTGGAAAG
GTTAGAACACGCATGTTAATTGACGATGAGATAGAGCCTCAAAGTTTTAGCACCCACACCACAATCCATGCTTAT
TTGGTGAATAGTGGCACGAAGAAAGTTCTTAAGGTTGATTTAACACCACACTCGTCGCTACGTGCGAGTGAGAAA
TCAACAGCAATAATGGGATTTCCTGAAAGGGAGAATGAATTGCGTCAAACCGGCATGGCAGTGCCAGTGGCTTAT
GATCAATTGCCACCAAGCGCATGGAGTCATCCTCAATTCGAGAAAGGTGGAGGTTCTGGCGGTGGATCGGGAGGT

TCAGCGTGGAGCCACCCGCAGTTCGAAAAATCCGGAAAGAGTGAGGACTTGACGTTTGAAGGAGAAAGCTTGTTT
AAGGGACCACGTGATTACAACCCGATATCGAGCACCATTTGTCACTTGACGAATGAATCTGATGGGCACACAACA
TCGTTGTATGGTATTGGATTTGGTCCCTTCATCATTACAAACAAGCACTTGTTTAGAAGAAATAATGGAACACTG
TTGGTCCAATCACTACATGGTGTATTCAAGGTCAAGAACACCACGACTTTGCAACAACACCTCATTGATGGGAGG
GACATGATAATTATTCGCATGCCTAAGGATTTCCCACCATTTCCTCAAAAGCTGAAATTTAGAGAGCCACAAAGG
GAAGAGCGCATATGTCTTGTGACAACCAACTTCCAAACTAAGAGCATGTCTAGCATGGTGTCAGACACTAGTTGC
ACATTCCCTTCATCTGATGGCATATTCTGGAAGCATTGGATTCAAACCAAGGATGGGCAGTGTGGCAGTCCATTA
GTATCAACTAGAGATGGGTTCATTGTTGGTATACACTCAGCATCGAATTTCACCAACACAAACAATTATTTCACA
AGCGTGCCGAAAAACTTCATGGAATTGTTGACAAATCAGGAGGCGCAGCAGTGGGTTAGTGGTTGGCGATTAAAT
GCTGACTCAGTATTGTGGGGGGGCCATAAAGTTTTCATGAGCAAACCTGAAGAGCCTTTTCAGCCAGTTAAGGAA
GCGACTCAACTCATGAGTGAATTGGTGTACTCGCAA

>TSTNIa3
GGGAAGAAGAATCAGAAGCACAAGCTTAAGATGAGAGAGGCGCGTGGGGCTAGAGGGCAATATGAGGTTGCAGCG
GAGCCAGAGGCGCTAGAACATTACTTTGGAAGCGCATATAATAACAAAGGAAAGCGCAAGGGCACCACGAGAGGA
ATGGGTGCAAAGTCTCGGAAATTCATAAACATGTATGGGTTTGATCCAACTGATTTTTCATACATTAGGTTTGTG
GATCCATTGACAGGTCACACTATTGATGAGTCCACAAACGCACCTATTGATTTAGTGCAGCATGAGTTTGGAAAG
GTTAGAACACGCATGTTAATTGACGATGAGATAGAGCCTCAAAGTTTTAGCACCCACACCACAATCCATGCTTAT
TTGGTGAATAGTGGCACGAAGAAAGTTCTTAAGGTTGATTTAACACCACACTCGTCGCTACGTGCGAGTGAGAAA
TCAACAGCAATAATGGGATTTCCTGAAAGGGAGAATGAATTGCGTCAAACCGGCATGGCAGTGCCAGTGGCTTAT
GATCAATTGCCACCAAAGAGTGAGGACTTGACGTTTGAAGGAGAAAGCAGCGCATGGAGTCATCCTCAATTCGAG
AAAGGTGGAGGTTCTGGCGGTGGATCGGGAGGTTCAGCGTGGAGCCACCCGCAGTTCGAAAAATCCGGA*GGAGAG*
*AGC*TTGTTTAAGGGACCACGTGATTACAACCCGATATCGAGCACCATTTGTCACTTGACGAATGAATCTGATGGG
CACACAACATCGTTGTATGGTATTGGATTTGGTCCCTTCATCATTACAAACAAGCACTTGTTTAGAAGAAATAAT
GGAACACTGTTGGTCCAATCACTACATGGTGTATTCAAGGTCAAGAACACCACGACTTTGCAACAACACCTCATT
GATGGGAGGGACATGATAATTATTCGCATGCCTAAGGATTTCCCACCATTTCCTCAAAAGCTGAAATTTAGAGAG
CCACAAAGGGAAGAGCGCATATGTCTTGTGACAACCAACTTCCAAACTAAGAGCATGTCTAGCATGGTGTCAGAC
ACTAGTTGCACATTCCCTTCATCTGATGGCATATTCTGGAAGCATTGGATTCAAACCAAGGATGGGCAGTGTGGC
AGTCCATTAGTATCAACTAGAGATGGGTTCATTGTTGGTATACACTCAGCATCGAATTTCACCAACACAAACAAT
TATTTCACAAGCGTGCCGAAAAACTTCATGGAATTGTTGACAAATCAGGAGGCGCAGCAGTGGGTTAGTGGTTGG
CGATTAAATGCTGACTCAGTATTGTGGGGGGGCCATAAAGTTTTCATGAGCAAACCTGAAGAGCCTTTTCAGCCA
GTTAAGGAAGCGACTCAACTCATGAGTGAATTGGTGTACTCGCAA

>TSTNIa4
GGGAAGAAGAATCAGAAGCACAAGCTTAAGATGAGAGAGGCGCGTGGGGCTAGAGGGCAATATGAGGTTGCAGCG
GAGCCAGAGGCGCTAGAACATTACTTTGGAAGCGCATATAATAACAAAGGAAAGCGCAAGGGCACCACGAGAGGA
ATGGGTGCAAAGTCTCGGAAATTCATAAACATGTATGGGTTTGATCCAACTGATTTTTCATACATTAGGTTTGTG
GATCCATTGACAGGTCACACTATTGATGAGTCCACAAACGCACCTATTGATTTAGTGCAGCATGAGTTTGGAAAG
GTTAGAACACGCATGTTAATTGACGATGAGATAGAGCCTCAAAGTTTTAGCACCCACACCACAATCCATGCTTAT
TTGGTGAATAGTGGCACGAAGAAAGTTCTTAAGGTTGATTTAACACCACACTCGTCGCTACGTGCGAGTGAGAAA
TCAACAGCAATAATGGGATTTCCTGAAAGGGAGAATGAATTGCGTCAAACCGGCATGGCAGTGCCAGTGGCTTAT
GATCAATTGCCACCAAAGAGTGAGGACTTGACGTTTGAAGGAGAAAGCTTGTTTAAGGGACCACGTGATTACAAC
CCGATATCGAGCACCATTTGTCACTTGACGAATGAATCTGATGGGCACACAACATCGTTGTATGGTATTGGATTT
GGTCCCTTCATCATTACAAACAAGCACTTGTTTAGAAGAAATAATGGAACACTGTTGGTCCAATCACTACATGGT
GTATTCAAGGTCAAGAACACCACGACTTTGCAACAACACCTCATTGATGGGAGGGACATGATAATTATTCGCATG
CCTAAGGATTTCCCACCATTTCCTCAAAAGCTGAAATTTAGAGAGCCACAAAGGGAAGAGCGCATATGTCTTGTG
ACAACCAACTTCCAAACTAAGAGCATGTCTAGCATGGTGTCAGACACTAGTTGCACATTCCCTTCATCTGATGGC
ATATTCTGGAAGCATTGGATTCAAACCAAGGATGGGCAGTGTGGCAGTCCATTAGTATCAACTAGAGATGGGTTC
ATTGTTGGTATACACTCAGCATCGAATTTCACCAACACAAACAATTATTTCACAAGCGTGCCGAAAAACTTCATG
GAATTGTTGACAAATCAGGAGGCGCAGCAGTGGGTTAGTGGTTGGCGATTAAATGCTGACTCAGTATTGTGGAGC
GCATGGAGTCATCCTCAATTCGAGAAAGGTGGAGGTTCTGGCGGTGGATCGGGAGGTTCAGCGTGGAGCCACCCG
CAGTTCGAAAAATCCGGAGGGGGCCATAAAGTTTTCATGAGCAAACCTGAAGAGCCTTTTCAGCCAGTTAAGGAA
GCGACTCAACTCATGAGTGAATTGGTGTACTCGCAA

>TSTNIa5
GGGAAGAAGAATCAGAAGCACAAGCTTAAGATGAGAGAGGCGCGTGGGGCTAGAGGGCAATATGAGGTTGCAGCG
GAGCCAGAGGCGCTAGAACATTACTTTGGAAGCGCATATAATAACAAAGGAAAGCGCAAGGGCACCACGAGAGGA
ATGGGTGCAAAGTCTCGGAAATTCATAAACATGTATGGGTTTGATCCAACTGATTTTTCATACATTAGGTTTGTG
GATCCATTGACAGGTCACACTATTGATGAGTCCACAAACGCACCTATTGATTTAGTGCAGCATGAGTTTGGAAAG
GTTAGAACACGCATGTTAATTGACGATGAGATAGAGCCTCAAAGTTTTAGCACCCACACCACAATCCATGCTTAT
TTGGTGAATAGTGGCACGAAGAAAGTTCTTAAGGTTGATTTAACACCACACTCGTCGCTACGTGCGAGTGAGAAA

**TCAACAGCAATAATGGGATTTCCTGAAAGGGAGAATGAATTGCGTCAAACCGGCATGGCAGTGCCAGTGGCTTAT
GATCAATTGCCACCAAAGAGTGAGGACTTGACGTTTGAAGGAGAAAGCTTGTTTAAGGGACCACGTGATTACAAC
CCGATATCGAGCACCATTTGTCACTTGACGAATGAATCTGATGGGCACACAACATCGTTGTATGGTATTGGATTT
GGTCCCTTCATCATTACAAACAAGCACTTGTTTAGAAGAAATAATGGAACACTGTTGGTCCAATCACTACATGGT
GTATTCAAGGTCAAGAACACCACGACTTTGCAACAACACCTCATTGATGGGAGGGACATGATAATTATTCGCATG
CCTAAGGATTTCCCACCATTTCCTCAAAAGCTGAAATTTAGAGAGCCACAAAGGGAAGAGCGCATATGTCTTGTG
ACAACCAACTTCCAAACTAAGAGCATGTCTAGCATGGTGTCAGACACTAGTTGCACATTCCCTTCATCTGATGGC
ATATTCTGGAAGCATTGGATTCAAACCAAGGATGGGCAGTGTGGCAGTCCATTAGTATCAACTAGAGATGGGTTC
ATTGTTGGTATACACTCAGCATCGAATTTCACCAACACAAACAATTATTTCACAAGCGTGCCGAAAAACTTCATG
GAATTGTTGACAAATCAGGAGGCGCAGCAGTGGGTTAGTGGTTGGCGATTAAATGCTGACTCAGTATTGTGGGGG
GGCCATAAAGTTTTCATGAGCAAACCTGAAGAGCCTTTTCAGCCAGTTAAGGAAGCGACTCAACTCAGCGCATGG
AGTCATCCTCAATTCGAGAAAGGTGGAGGTTCTGGCGGTGGATCGGGAGGTTCAGCGTGGAGCCACCCGCAGTTC
GAAAAATCCGGAATGAGTGAATTGGTGTACTCGCAA**
