# Supplementary figures and images for: Interaction network of tobacco etch potyvirus NIa protein with the host proteome during infection
Source: BMC Genomics. 2016 Feb 1;17:87. doi: 10.1186/s12864-016-2394-y (PMC4735970; doi:10.1186/s12864-016-2394-y)

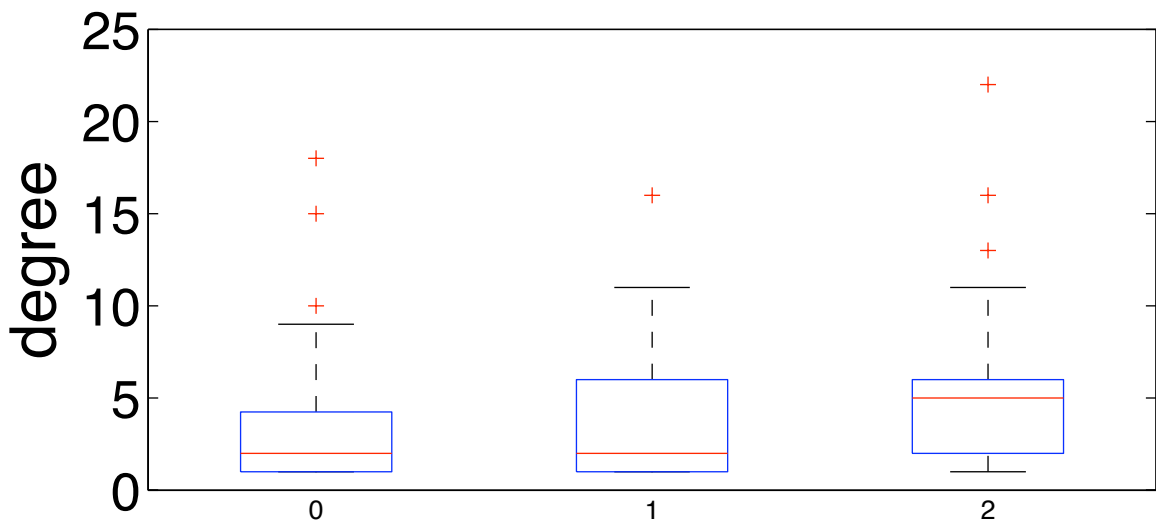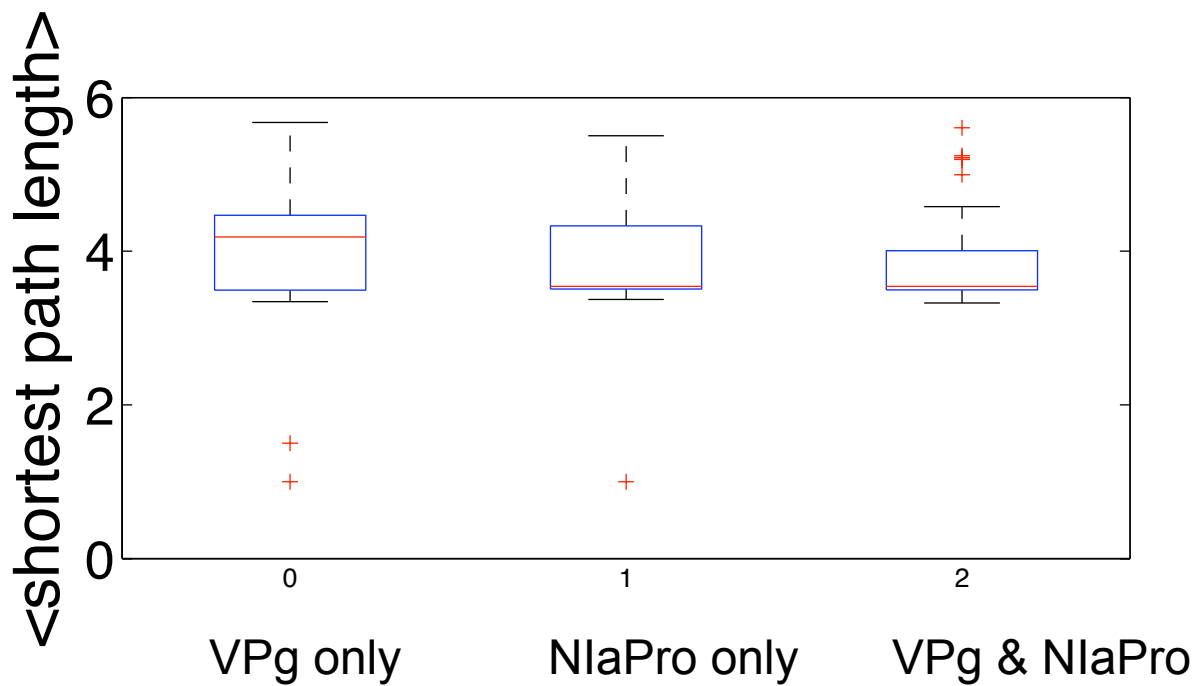

Supplement: Additional file 5: — Boxplot of the global topological properties (degree and average shortest path length) of the NIa targets covered in the experimental A. thaliana interactome. Note that these properties were calculated according to the global interactome and not to a particular subnetwok. No significant differences were found between distributions, except for the comparison in degree between specific VPg targets and those in common (Mann–Whitney U-test, P = 0.03). (PDF 21 kb) [file 12864_2016_2394_MOESM5_ESM.pdf]

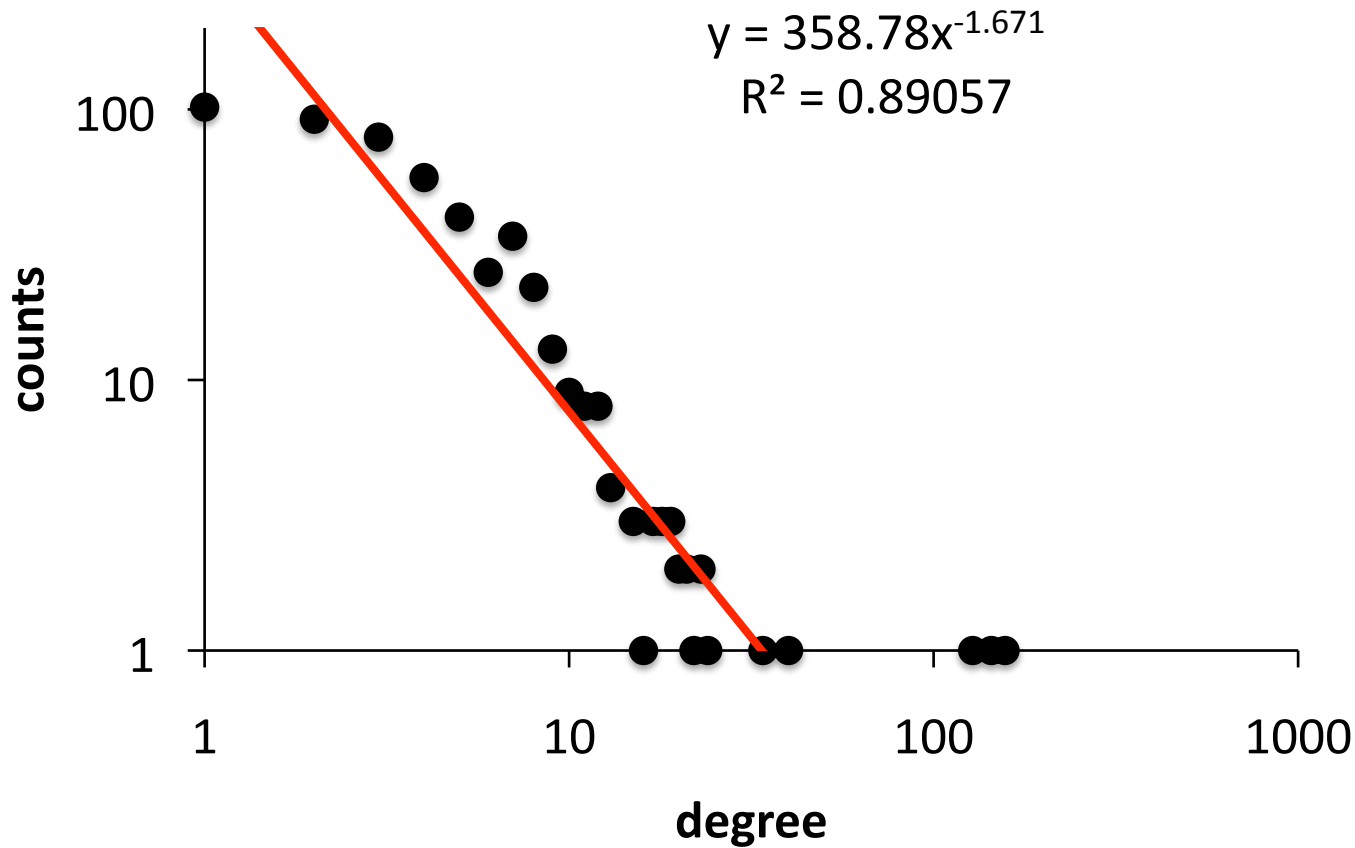

Supplement: Additional file 6: — Degree distribution of the NIa network showing a scale-free trend. The three outliers with higher degree were not considered in the regression. (PDF 38 kb) [file 12864_2016_2394_MOESM6_ESM.pdf]
